# Development of Semi-Automated Image-Based Analysis Tool for CBCT Evaluation of Alveolar Ridge Changes After Tooth Extraction

**DOI:** 10.3390/bioengineering12030307

**Published:** 2025-03-18

**Authors:** Anja Heselich, Joanna Śmieszek-Wilczewska, Louisa Boyo, Robert Sader, Shahram Ghanaati

**Affiliations:** 1 FORM-Lab, Department for Oral, Cranio-Maxillofacial and Facial Plastic Surgery, Medical Center of the Goethe University Frankfurt, Goethe University, 60590 Frankfurt am Main, Germanyr.sader@em.uni-frankfurt.de (R.S.);; 2 Denticus Clinic, Lelewela 1/1, 44-100 Gliwice, Poland

**Keywords:** socket preservation, alveolar ridge preservation, bone regeneration, atrophy, radiological evaluation

## Abstract

Following tooth extraction, the bone structure is prone to atrophic changes. Alveolar ridge resorption can compromise subsequent implant treatment not only at the extraction site itself but also by affecting the bone support of adjacent teeth. Various techniques, including the use of bone graft materials or autologous blood concentrates for ridge or socket preservation, aim to counteract this process. The efficacy of such methods can be evaluated non-invasively through radiological analysis of the treated region. However, existing radiological evaluation methods often focus only on isolated areas of the extraction socket, limiting their accuracy in assessing overall bone regeneration. This study introduces a novel, non-invasive, and semi-automated image-based analysis method that enables a more comprehensive evaluation of bone preservation using CBCT data. Developed with the open-source software “Fiji” (v2.15.0; based on ImageJ), the approach assesses bone changes at multiple horizontal and vertical positions, creating a near three-dimensional representation of the resorptive process. By analyzing the entire region around the extraction socket rather than selected regions, this method provides a more precise and reproducible assessment of alveolar ridge preservation. Although the approach requires some processing time and focuses exclusively on radiological evaluation, it offers greater accuracy than conventional methods. Its standardized and objective nature makes it a valuable tool for clinical research, facilitating more reliable comparisons of different socket preservation strategies.

## 1. Introduction

Tooth extraction leads to changes not only within the extraction socket but also in surrounding anatomical structures, including bone, soft tissue, and adjacent teeth. Initially, the loss of the bundle bone occurs as it is no longer functionally required [1]. During the bone modeling phase of alveolar healing, additional bone resorption follows, leading to reductions in both ridge width and height [2]. Bone atrophy is likely due to the loss of intraosseous stimulation by the tooth, with additional contributing factors such as reduced blood supply and localized inflammation [1].

**Figure 1:**
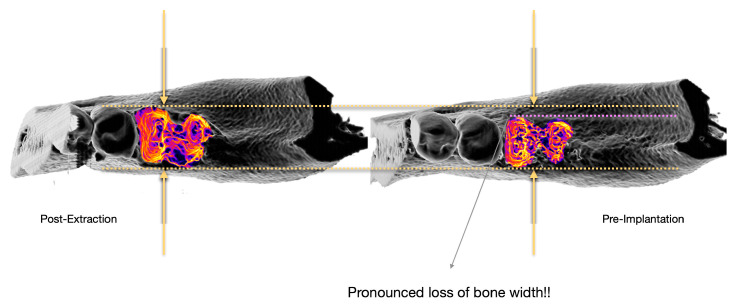
Visible changes in bone dimension during bone regeneration post-tooth extraction. Comparison of the situation post-tooth extraction (**left**) to the bone dimensions after a certain regeneration time (**right**, “pre-implantation”) reveals a pronounced loss of bone width.

The healing of the alveolar socket is a complex biological process described in three phases: inflammatory, proliferative, and regenerative [2,3,4,5]. While originally deemed to be similar to long bone healing, recent research suggests that alveolar bone remodeling follows a distinct pattern. Instead of new bone forming inward from the lateral walls, post-extraction bone loss is primarily driven by the collapse of the alveolar walls toward the socket center, with peripheral bone apposition failing to restore the original volume. Soft tissue closure occurs crestally, but residual voids may persist in deeper regions [6]. These structural changes significantly impact ridge dimensions (for an example, see Figure 1), particularly affecting vestibular bone and contributing to both vertical and horizontal resorption [7,8,9,10].

Bone resorption progresses rapidly post-extraction, with two-thirds of the bone loss occurring within the first three months [10]. Thereafter, a slower, lifelong resorption continues [1]. The lack of mechanical stimulation and the structural properties of bundle bone exacerbate this process [11,12]. Studies indicate greater bone loss buccally than orally [13,14], more in the mandible than the maxilla [10], and higher losses in molars than in premolars [10,14]. A systematic review by Van der Weijden et al. [15] analyzed ridge dimensional changes following extraction with natural healing over 3–12 months. A meta-analysis of six studies revealed an average clinical ridge width reduction of 3.87 mm and an approximated bone level loss of 0.64 mm. Radiographic analysis in six additional studies, including molars, indicated a 1.21 mm reduction in ridge width and a 0.7 mm reduction in bone height.

Since these changes pose risks not only for future implant success but also for adjacent tooth stability [6], strategies to minimize post-extraction bone loss are critically important.

To support bone formation and minimize resorption post-extraction, biomaterials can be placed within the extraction socket. Various techniques exist for this purpose. The placement of bone graft materials in the socket, termed socket preservation or alveolar ridge preservation, aims to maintain alveolar ridge dimensions [16]. While the terms are often used interchangeably, socket preservation is typically performed when bony walls remain intact, whereas ridge preservation applies when one or more socket walls are compromised [17]. A variety of biomaterials, including bone grafts and collagen membranes, have been investigated for their effectiveness in maintaining ridge volume [17,18,19,20,21]. Additionally, autologous blood concentrates such as Platelet-Rich Fibrin (PRF) have been explored as standalone treatments or in combination with grafting materials to promote healing and bone regeneration [21,22,23,24,25,26,27,28,29].

The graft material serves as a scaffold for vascular ingrowth and new bone formation, a process known as osteoconduction [16]. Another technique, the socket seal, involves covering the socket with a barrier membrane to protect regenerating bone, prevent soft tissue invasion, and guide healing [30]. This technique is particularly beneficial in cases with compromised bony walls [31]. Socket preservation and socket seal may be applied independently or in combination. Their advantage lies in being performed immediately during extraction, reducing the need for later bone augmentation [30].

A meta-analysis by Avila-Ortiz et al. [32] demonstrated that socket preservation significantly mitigates ridge width and height loss compared to spontaneous healing without biomaterials. The need for additional augmentation prior to or during implantation was lower in cases with socket preservation. This meta-analysis included randomized clinical trials employing various biomaterials, with or without socket seal, over healing periods of 3–6 months. While the authors recommended socket preservation to limit post-extraction bone loss, no single biomaterial proved superior for this indication.

Accurate evaluation of post-extraction ridge changes is essential for assessing intervention success. Various clinical and radiographic methods, including calipers, customized periodontal probes, and Cone Beam Computed Tomography (CBCT), have been used to measure alveolar ridge dimensions [25,26]. CBCT-based methods have become standard for assessing three-dimensional bone loss. However, most existing techniques focus on limited ridge areas. Some studies measure vertical and horizontal bone loss in axial and sagittal CBCT slices [23,28,29,33,34,35,36], while others assess bone density through grey value analysis at specific central socket points [34,37]. A more recent approach by Abellán et al. [38] introduced measurements at three depths but only in a single sectional plane, potentially overlooking ridge-wide variations.

Given the complexity of alveolar ridge remodeling, a more detailed and standardized method is needed for comprehensive evaluation. Many current approaches focus on crestal changes or rely on single-plane analysis, limiting their three-dimensional scope [2,7,9,10,12,23,36,38]. This study aimed to develop a semi-automated image-based method for the precise and reproducible assessment of post-extraction ridge alterations. The proposed method enables measurements at five distinct positions around the socket in the horizontal plane and at three depths, from the crestal level to 5 mm below. By implementing a structured, multi-dimensional approach, this technique provides a more reliable assessment of bone resorption and facilitates objective comparisons between different ridge preservation strategies.

## 2. Materials and Methods

### 2.1. Software and Data Format

Image-based evaluation of bone change was performed in imaging software Fiji (Fiji Is Just ImageJ, v2.15.0). Fiji is built on ImageJ2 (v1.54g), an advanced version of the scientific and medical image analysis software ImageJ provided by the National Institutes of Health (NIH, Bethesda, MD, USA; imagej.nih.gov, accessed on 14 May 2024) [39]. All processing and analyzing tools used and implemented in the analysis macro are included in Fiji.

The 3D Cone Beam Computed Tomography (CBCT) images used for analysis were recorded and the resulting files in the Digital Imaging and Communications in Medicine (DICOM) format were analyzed.

### 2.2. Clinical Trial Study Design

For method development, CBCTs of the control group of the following randomized, controlled, clinical trial were evaluated.

#### 2.2.1. Study Design

The clinical trial was a prospective, parallel-arm, randomized, controlled trial (RCT) conducted at the Medical University of Silesia, Katowice, Poland, from January 2018 to May 2022. This study followed the Declaration of Helsinki and national regulations in Poland and Germany for human studies.

Surgical interventions and follow-ups, performed by SG and JS, were approved by the Ethics Committee of the Faculty of Medicine at Goethe University, University Hospital Frankfurt/Main, Germany (#19/227; 2019/10/26). After tooth extraction sockets were either left to natural healing (control group, unfilled and untreated socket) or filled with bone substitute material for socket preservation (treatment group, data are not part of this publication). Overall, the aim of the RCT was to assess the efficacy of bone regeneration and preservation. All participants provided written informed consent after being informed of this study’s procedures and objectives.

#### 2.2.2. Inclusion and Exclusion Criteria

Patients aged 18 years and older who required extractions of premolars and were scheduled for dental implant therapy were enrolled in this study. Detailed medical and dental histories were collected. Exclusion criteria encompassed individuals who did not provide written consent, were pregnant, had uncontrolled metabolic disorders, had untreated periodontal disease, exhibited periapical lesions, were at risk for bisphosphonate-related MRONJ, had poor oral hygiene, or were unable to comply with study protocols. Additionally, patients with root fractures, a risk of residual fragments, or pre-existing alveolar bone defects were excluded from participation.

### 2.3. Sample Size Calculation

A priori power analysis was conducted to determine the required sample size for a two-tailed *t*-test based on the primary endpoint, bone volume. Sample size calculation was conducted with G*Power 3.1 power calculation software provided by the Heinrich-Heine-University Düsseldorf, Düsseldorf, Germany [40]. Given an effect size (d) of 1.019, an alpha level (α) of 0.05, and a desired power (1-β) of 0.80, the analysis determined a required sample size of *n* = 17 patients per group. To account for potential dropouts, recruitment of *n* = 18 patients per group was planned.

### 2.4. Surgical Procedures

#### 2.4.1. Tooth Extraction and Socket Preservation

The extractions were carried out under local anesthesia using a minimally invasive technique, without the need for vertical release incisions. To reduce the risk of root and bone fractures, teeth were, if necessary, sectioned using the piezo technique. The sockets were then inspected, curetted, and irrigated with sterile 0.9% saline. In the control group, sockets were left to natural healing (untreated and unfilled). In the treatment groups, socket preservation with bovine bone substitute material was performed. The dataset of the latter group is not part of the current manuscript.

#### 2.4.2. Follow-Up and Radiological Evaluation

Tooth extraction and treatment were evaluated for bone condition and regeneration on day 0 and day 180 post-extraction. The primary outcome was the change in bone dimensions six months after extraction. CBCT imaging was performed immediately after extraction and again before implantation at the six-month mark. CBCTs were taken using a CS8100 3D CBCT scanner (Carestream Dental) with a field of view (FOV) of 8 × 5 or 8 × 9 mm, depending on the region scanned, and a slice thickness of 0.15 mm. The scanned images were subjected to the reconstruction process and clinical evaluation using CS 3D Imaging (Carestream Dental) software (version 3.10.21.0). For further processing and evaluation in Fiji, DICOM file sets were extracted using the same software.

Bone dimensions were assessed at both time points. Prior to analysis, the CBCT scans underwent a qualitative review, and those with optical artifacts, such as overexposed areas caused by adjacent prosthetic restorations or incomplete alveolar images, were excluded to ensure accurate and reliable assessments. After a quality review, CBCTs of *n* = 10 patients from the control group were ultimately included in the evaluation.

### 2.5. Statistical Analysis

Descriptive statistics and statistical analyses were performed using GraphPad Prism software (version 10.3.1, GraphPad Software, LLC, San Diego, CA, USA). Bone dimensional changes were analyzed using one-way ANOVA with Tukey’s multiple comparisons post-hoc test at a 95% confidence interval (CI). The reproducibility of measurements was assessed using the Intraclass Correlation Coefficient (ICC) and Bland–Altman analysis, with data analysis performed in RStudio (v2024.12.1) using the psych package. The ICC was calculated using a mixed-effects model to evaluate both inter-rater and intra-rater reliability across probes and positions. Bland–Altman analysis was used to plot the differences between the measurements of two raters against the mean of the two measurements. The limits of agreement, calculated as the mean difference ± 1.96 times the standard deviation of the differences, were determined, and analysis was checked for outliers were identified.

## 3. Results

### 3.1. Method Development

**Figure 2:**
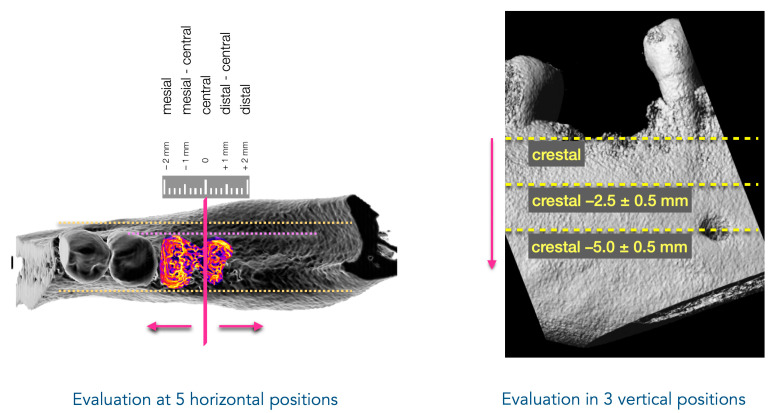
Evaluation positions of the novel image-based analysis method for bone dimensional change. Bone dimensions were evaluated at 5 horizontal positions: central, ±1 mm, and ±2 mm in the mesial and distal directions (**left**). Additionally, 3 vertical positions were assessed: crestal, −2.5 mm, and −5 mm below the crestal level (**right**). Depending on the available CBCT slice dimensions, vertical positions could be defined with an accuracy of ±0.5 mm.

A semi-automated image analysis technique was developed to evaluate bone regeneration in extraction sockets, utilizing a step-by-step processing approach of CBCT images. Changes in bone dimension, specifically the width of the alveolar ridge at 15 positions (five horizontal, each at three vertical depths: crestal, 2.5 mm, and 5 mm below the crestal level, Figure 2), were analyzed. The focus was on the efficacy of the chosen treatment for the preservation of bone dimensions during the regeneration process. For this, DICOM raw data from the CBCT scans at two time points, directly after tooth extraction and after a regeneration period of, e.g., 3 months, were processed to prepare for analysis.

#### 3.1.1. CBCT Preparation and Processing for Measurement Analysis

**Figure 3:**
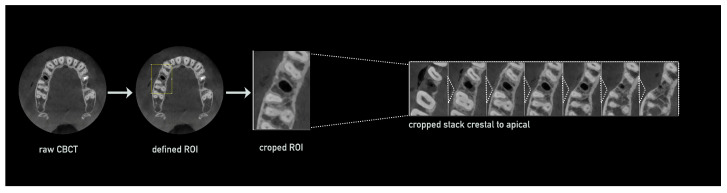
Semi-automated CBCT processing in preparation for bone width evaluation. The raw CBCT data were imported, followed by the definition of the region of interest (ROI) and cropping to isolate the area including the treated socket. The resulting stack encompassed the entire region from the crestal to the apical aspect.

The first step was the optimization of the available CBCT scans for smoother processing via cropping to the region of interest including the area of extraction: DICOM raw data were imported into Fiji using the Bio-Formats Importer plugin. The greyscale range was standardized to 8 bit and saved in TIFF format for easier processing. The voxel size was verified and, if compressed, readjusted to the original dimensions. The Slice Keeper tool was then used to remove irrelevant apical and coronal areas, such as the opposing jaw, or parts of the maxillary sinus, while retaining slices containing the empty socket and adjacent teeth. The region of interest was further refined by defining a rectangular area around the socket and adjacent teeth in the axial slice using the Rectangular tool and cropping the CBCT stack in all dimensions (apical–coronal, vestibular–oral, and mesial–distal; Figure 3). The cropped stack was then rotated to align the socket and adjacent teeth along a vertical axis.

To ensure consistent processing of the second CBCT taken after the regeneration period, the rotated and cropped stack from the first CBCT (post-extraction) was used as a reference. After preprocessing, both CBCT stacks were aligned to ensure identical stacks with minimal rotational discrepancies for subsequent measurements.

#### 3.1.2. Definition of the Measurement Positions

**Figure 4:**
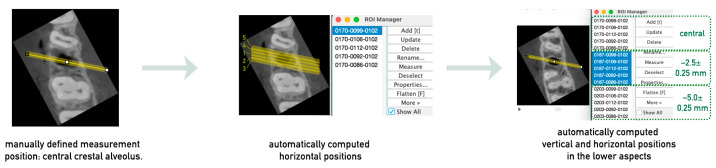
Definition of measurement positions. The reference position, at the center of the extraction socket in the crestal plane (**left**), was defined manually. Based on its coordinates, the five positions in the horizontal plane (**middle**) and further positions in the lower aspects (**right**) were calculated automatically.

To measure the alveolar ridge width initially at the crestal edge, the slice containing this reference point was selected. Since regeneration can alter both the width and height of the alveolar ridge, the starting slice for measurements was chosen based on the second CBCT (post-regeneration) to ensure that measurements were taken at the same axial level in both CBCTs. The Orthogonal Views function was used to facilitate orientation by displaying sagittal and coronal views alongside the axial view. After identifying the crestal slice in the second CBCT and selecting the corresponding slice in the first CBCT, the semi-automated definition of the measurement positions could be provided. A straight line with a line width of at least 10 was drawn perpendicular to the alveolar ridge in the center of the alveolus in the first CBCT, extending beyond the ridge width. This line, representing the central–crestal evaluation position, was added to the ROI Manager. Additional measurement lines were automatically generated and added using a macro code, which duplicated and shifted the central line by defined distances (1 mm and 2 mm mesially and distally). In the next step, the measurement positions at different axial levels were defined (Figure 4). Based on the first measurement plane, which corresponded to the crestal edge of the alveolar ridge, two additional measurement planes were established at 2.5 ± 0.25 mm and 5.0 ± 0.25 mm apical to the crestal plane. The correct slices for these planes were selected by determining the number of slices corresponding to 2.5 mm and 5.0 mm, considering the CBCT slice thickness, with a tolerance of ±0.25 mm to ensure comparability. After defining the correct measurement planes, the measurement positions were transferred to the two deeper planes and stored in the ROI Manager. This resulted in a total of 15 measurement positions.

#### 3.1.3. Evaluation of Alveolar Crest Width at the Measurement Positions

**Figure 5:**
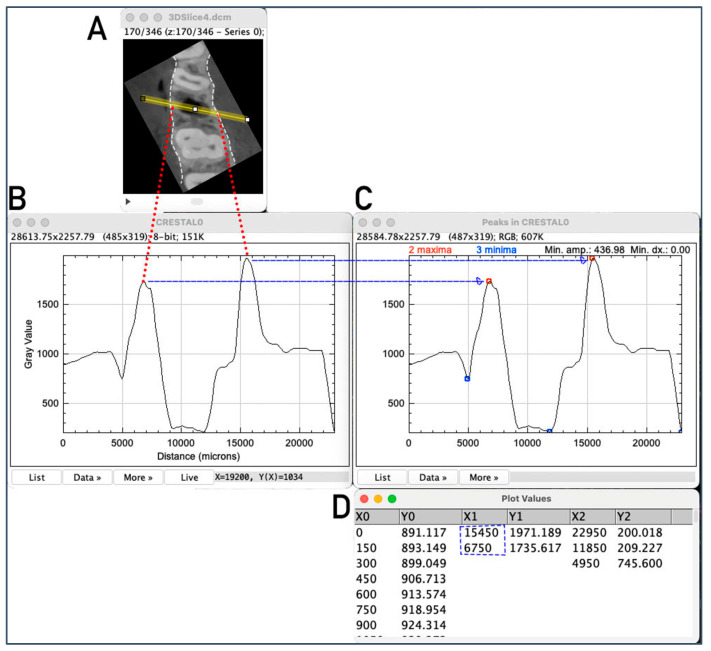
Evaluation of the alveolar crest width via peak definition of grey value line-plot. Example of alveolar crest width measurement at the central–crestal position. A mean grey value plot along the measurement line (**A**) was created, with peaks indicating the grey value maxima (**B**), which corresponded to the outer rim of the alveolar crest (highlighted by dashed lines in (**A**). The line plot was automatically analyzed using the Peak Finder plug-in (**C**), and peak coordinates were directly obtained from the provided plot data (**D**). The difference between the x-coordinates of the maxima determined the alveolar crest width at this position.

For the measurement of the alveolar crest width, the first measurement position in the ROI Manager was selected, and a grey value plot was generated using the Plot Profile function. The x-axis of the profile plot represented the length of the line in millimeters and the y-axis displayed the grey value ranging from 0 to 256 (Figure 5B). The Find Peaks plugin, with the List values setting, could then be applied to directly mark the minima and maxima of the graph. A second graph was generated showing the minima as blue and the maxima as red points (Figure 5C). The corresponding list with the x- and y-coordinates included coordinates of the maxima as well (Figure 5D). The difference in the x-values of the maxima represented the corresponding alveolar crest width. This procedure was automatically repeated for all 15 positions to determine the corresponding alveolar crest width. The measurement was performed analogously for the second CBCT taken after a defined regeneration period. The difference between the two time points indicated the dimensional change of the alveolar crest, showing the extent of ridge preservation at each position.

### 3.2. Reproducibility Analysis

For the evaluation of the reproducibility of the measurements, six CBCT scans were analyzed three times by two individual raters, resulting in 15 evaluation positions per CBCT scan. The evaluations were performed in repeated consecutive rounds, where the entire set was analyzed, and then the next round of evaluations was started. The reproducibility of the measurements and the resulting data were assessed using the Intraclass Correlation Coefficient (ICC) and Bland–Altman analysis.

The results of the ICC analysis performed in RStudio showed excellent reliability. For inter-rater reliability, the ICC values for single random and single fixed raters were 0.99, indicating almost perfect agreement between raters. When averaging across measurements, the ICC even reached 1.00. Intra-rater reliability was also high, with Rater 1 showing an ICC of 0.994 and Rater 2 an ICC of 0.988, both indicating excellent consistency within individual raters (*p* < 0.001). The ICC results were supported by the Bland–Altman analysis, which revealed a mean difference of 0, indicating no significant bias between the raters. The limits of agreement (CI = 95%) were 0.45 for the upper limit and −0.4 for the lower limit. Only very few outliers were identified, five in total, of which two were above the upper limit (approximately 0.5) and three were below the lower limit (approximately −0.5, −0.55, and −1.25).

### 3.3. Application of the Method to Clinical Data

**Figure 6:**
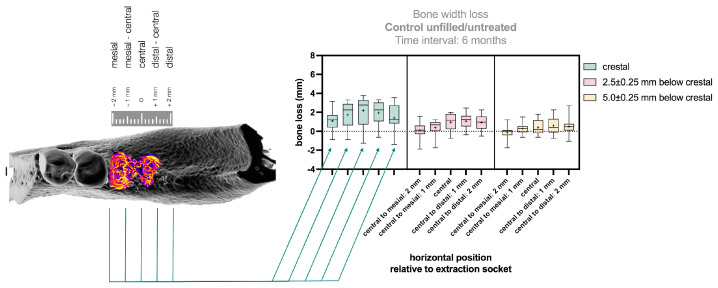
Evaluation of bone width after 6 months of natural healing post-tooth extraction. Bone changes were assessed at the crestal level (green) and 2.5 ± 0.5 mm (pink) and 5.0 ± 0.5 mm (yellow) below the crestal level. In each horizontal plane, bone dimensions were measured at the center of the extraction socket, as well as ±2 mm and ±4 mm in the mesial and distal directions. The results clearly show that bone loss occurred predominantly at the crestal level, with a less pronounced reduction at lower levels. Additionally, bone loss was most significant at the center of the alveolus, with diminishing values toward the peripheral regions. This pattern was still somewhat visible in the lower regions (pink, yellow). In the central evaluation position, the difference between the crestal level and the level 5.0 ± 0.25 mm below was statistically significant (*p* = 0.0224; CI = 95%). Plot presents a Box–Whisker–Blot ± Min/Max, line = median, + = mean; *n* = 10.

The alveolar crest width and its preservation were determined for control patients with extraction sockets in a controlled clinical trial (Figure 6). The greatest width loss occurred at the crestal measurement level compared to the other levels (Figure 6, green). The central position showed the greatest width loss (2.18 ± 1.61 mm). From this point, the loss decreased mesially (1 mm: 1.74 ± 1.48 mm; 2 mm: 1.10 ± 1.13 mm) and distally (1 mm: 1.95 ± 1.26 mm; 2 mm: 1.40 ± 1.38 mm), with the smallest bone loss at the 2 mm mesial position.

At the second measurement level, 2.5 ± 0.25 mm apical to the crestal plane (Figure 6, pink), the greatest width loss was observed centrally (0.97 ± 0.94 mm) and distally (1 mm: 1.08 ± 0.80 mm; 2 mm: 0.93 ± 0.84 mm). At the mesial position, the change in crest width was less pronounced (1 mm: 0.37 ± 0.93 mm; 2 mm: 0.12 ± 0.90 mm).

The third measurement level, 5.0 ± 0.25 mm apical to the crestal plane (Figure 6, yellow), showed the greatest width loss centrally (0.41 ± 0.80 mm) and distally (1 mm: 0.60 ± 0.98 mm; 2 mm: 0.52 ± 0.97 mm), with lower values at the mesial position (1 mm: 0.28 ± 0.62 mm; 2 mm: −0.14 ± 0.77 mm). No statistically significant differences were found between the various measurement positions across the three levels (see Supplementary Table S1), except in the central evaluation position, where a significant difference was observed between the crestal level and the level 5.0 ± 0.25 mm below (*p* = 0.0224, CI = 95%).

## 4. Discussion

Effective bone preservation and regeneration in post-extraction sockets is a key concern in dental practice, particularly when implant-based prosthetics are planned. While natural healing remains the traditional approach, socket or ridge preservation has become a widely used therapy, which typically involves the placement of biomaterials into the extraction site [17,18,19,20]. However, the non-invasive, semi-quantitative evaluation of bone regeneration remains a challenging task. Numerous methods have been proposed, but they often face issues with low compatibility and poor reproducibility, making it difficult to reliably assess the effectiveness of various socket and ridge preservation strategies. To overcome these challenges, this study aimed to develop a semi-automated, imaging-based technique that allows for reproducible, semi-quantitative assessments of bone preservation, specifically focusing on bone width in tooth extraction sockets using CBCT data. This method is intended to facilitate comparability, enabling consistent and quantitative comparisons of different preservation approaches. The newly developed image analysis technique was applied to evaluate bone regeneration and preservation in clinical trials for socket/ridge preservation. The first approach was to characterize bone preservation in extraction sockets during natural healing, both qualitatively and quantitatively, using data from a patient group within a trial that served as a control group with untreated, unfilled sockets.

### 4.1. Development of a Novel Evaluation Method and Application to Clinical Data

In the current literature on socket preservation, linear measurements are more commonly described for assessing changes in bone dimensions than volumetric measurements. These linear measurements also include the measurement of alveolar ridge width. The literature provides both clinical and radiographic data for these parameters. A significant advantage of clinical measurements is the simplicity of the examination, which can be performed intraorally, directly on the patient. Clinical measurements of ridge width or height reflect the dimensions of both the jawbone and the overlying soft tissue. For implant planning, soft tissue also plays an important role, especially in achieving aesthetically pleasing results [41]. However, when determining whether implantation is possible, the primary factor remains the hard tissue, i.e., the bone [42]. For this reason, usually, CBCT scans are used to evaluate bone availability and changes in bone dimensions to provide a deeper insight.

In this study, a novel, image-based analysis method using CBCTs was developed to assess ridge changes after extraction and regeneration over a defined healing period. Using this method, the alveolar ridge width was measured at five different positions from mesial to distal within the extraction socket. These five measurement points were taken at the crestal edge of the ridge, as well as 2.5 ± 0.25 mm and 5 ± 0.25 mm apical to the crestal measurement level. The semi-automated approach enabled a highly detailed and reproducible definition of the evaluation positions, providing a more comprehensive three-dimensional view of the region of interest.

Applying this method to evaluate ridge preservation after natural healing, the greatest reduction in ridge width was observed at the crestal measurement level. This finding aligns with general expectations and supports recent claims that a crestal collapse marks the initial stage of socket closure [6].

The greatest width loss during alveolar healing occurred in the coronal or crestal region of the socket, as clearly observed in the qualitative assessment of CBCT images from vestibular to oral. The two apical measurement levels exhibited minimal or no change in ridge width, with no statistically significant differences between them. The most pronounced width loss was recorded at the central part of the crestal level, with decreasing values toward the lateral sides in both mesial and distal directions, indicating better preservation in these areas. Evaluations at lower planes also showed width reduction, with the loss decreasing progressively at deeper levels. Nevertheless, the overall pattern remained consistent, with greater width loss centrally and better preservation laterally.

It has been well known for several decades that atrophic processes in the edentulous jaw lead to ongoing bone loss [11]. The recent literature evaluating changes in alveolar crest width during the course of alveolar healing without additional treatment continues to consistently indicate bone dimensional loss [24,28,35]. For example, Castro et al. [24] measured changes in the alveolar crest width one millimeter subcrestally, both vestibularly and orally, directly after extraction and three months post-extraction, finding a change of 2.2 ± 1.3 mm in the control group. Temmerman et al. [35] used the same methodology, excluding vestibularly compromised alveoli, and found a reduction of approximately 5.9 ± 5.2 mm in alveolar crest width in the control group. Similarly, results from Yewale et al. [28] were comparable in both method and findings. Comparable to Castro et al. [24], Canellas et al. [23] observed a clear loss of alveolar crest width in the control group of 2.27 ± 1.2 mm at one millimeter subcrestally in CBCT slices.

Another difference, besides the high number of evaluation points, between the measurements in this study and those in the literature is that the measurement described here was taken as far as the crest could be identified in the CBCT stack, while other studies measured one millimeter subcrestally. Nevertheless, the changes observed in these studies largely align with the results of this work, with a reduction of 3.75 mm (upper limit) and the highest mean loss of 2.18 ± 1.61 mm.

While the numerical differences appear in some cases substantial, they are overall largely consistent with previous findings. As in this study, the most significant loss in alveolar crest width was found in the central–crestal region. It can be assumed that size-dependent loss of the alveolus (e.g., premolar vs. molar [10,14]) is also at deeper levels, but this hypothesis requires further investigation. For a more precise evaluation of this and of socket preservation techniques overall, the detailed analysis provided by the method presented here will offer deeper insights in future trials.

### 4.2. Strengths and Limitations of Radiological Evaluation

The development of the method and its implementation as a macro code for Fiji to semi-automate the evaluation process has enhanced accessibility for both novice and experienced users. The method can be easily applied without extensive training, allowing for significantly faster analysis. The automated calculation of all regions for measurement, except for the initial positioning, minimizes user-induced bias in selecting evaluation areas. Reproducibility and transferability were tested by having two independent users evaluate the same set of CBCT images from a randomized controlled trial multiple times. The results demonstrated high consistency confirmed by ICC and Bland–Altman analyses, which demonstrated strong inter-rater and intra-rater reliability, with minimal systematic bias. While there were a few outliers, they did not detract from the overall consistency and reproducibility of the measurements.

Comparing the results gained with this novel method to the existing literature is challenging due to the variety of methodological approaches used. For example, Abellan et al. [38] used an approach that, like the current one, evaluates bone changes not just at the crestal level but also at three depths below it. While this method is efficient in terms of time and resources as it focuses only on the central region, it also has limitations since it excludes the peripheral aspects of the alveolus. In contrast, this study provides a comprehensive analysis of the entire socket area, offering a very accurate representation of bone dimension preservation. However, it is important to note that a standardized methodological framework is lacking in the literature, which still complicates overall comparisons of study results.

### 4.3. Clinical Relevance

This analytical tool can be effectively utilized to assess bone alterations and available bone volume prior to implant planning. By providing a precise understanding of the osseous conditions, it facilitates a more comprehensive preoperative evaluation, allowing clinicians to determine whether simultaneous bone augmentation is required or if a staged bone grafting procedure should be considered in advance. Considering that the lower regions in the present analysis were less compromised in terms of bone dimensional loss, this information should be taken into account when focusing on the preservation of the crestal region, which exhibits significantly more atrophic features. This approach helps avoid the common practice of placing the implant solely in the region with the seemingly best bone quality to achieve primary stability—a necessity that may compromise optimal physiological positioning. Instead, the knowledge gained through this tool, along with potential adjustments in treatment planning, such as additional augmentation or other preparatory measures, can create improved conditions for placing the implant in the most anatomically and biomechanically favorable location, ideally where the original tooth was positioned. However, the application of this tool requires the availability of CBCT imaging, at a minimum shortly before the planned implantation, but ideally also as a comparative scan following tooth extraction. Optimizing implant placement in this manner ensures that occlusal forces are transmitted along a more natural vector to the jawbone, thereby reducing the risk of mechanical complications and implant failure.

The novel method described here can be highly valuable for the long-term monitoring of alveolar ridge changes through non-invasive radiological analysis, enabling comparative assessments of different socket preservation techniques and providing insights into their efficacy over time. Combined with our recently published, newly developed method for the volumetric assessment of extraction sockets, this approach offers a comprehensive framework for evaluating alveolar ridge preservation strategies [43].

## 5. Conclusions

The developed method offers higher precision compared to many existing approaches, allowing for a more comprehensive assessment of bone preservation. However, it is technically demanding and relies on the quality of raw CBCT data. In contrast, simpler, time-efficient methods in the literature provide less detailed results, often focusing on only part of the relevant region. In application to clinical data, our method showed significant bone loss at the alveolar crest in sockets without further preservation, consistent with existing knowledge. Given the variety of methods and the need for clearer data, further studies using this approach—considering the entire socket—are essential for evaluating the effectiveness of socket and ridge preservation techniques. Recent findings suggest that alveolar healing involves both wall remodeling and peripheral appositional bone deposition, a notion supported by our results. Future studies should account for these structural changes to distinguish true bone regeneration from morphological adaptations, providing more definitive insights into alveolar regeneration.

## Data Availability

Data is contained within the article or Supplementary Materials.

## Supplementary Materials

The following supporting information can be downloaded at https://www.mdpi.com/article/10.3390/bioengineering12030307/s1: Table S1: Tukey’s multiple comparison post-hoc test results. Comparison of changes between different levels at the same horizontal evaluation position. (Evaluation conducted with GraphPad Prism).

## Abbreviations

The following abbreviations are used in this manuscript:

PRF	Platelet-Rich Fibrin
CBCT	Cone Beam Computer Tomography
