# Supplementary material for: Development of Semi-Automated Image-Based Analysis Tool for CBCT Evaluation of Alveolar Ridge Changes After Tooth Extraction
Source: Bioengineering (Basel). 2025 Mar 18;12(3):307. doi: 10.3390/bioengineering12030307 (PMC11939539; doi:10.3390/bioengineering12030307)
Supplement: Supplementary file 1 [file bioengineering-12-00307-s001.zip › bioengineering-3505547-supplementary.pdf]

Supplementary File

Table S1: Tukey's multiple comparison post-hoc test results. Comparison of changes between different levels at the same horizontal evaluation position. (Evaluation conducted with GraphPad Prism.)

Groups	*Mean 1*	*Mean 2*	*Mean Diff.*	*n1*	*n2*	*q*	*DF*	*95% CI of diff.*	*Adjusted P Value*
Crestal Mesial 2 mm vs. 2.5 mm Mesial 2 mm	1,096	0,1191	0,9765	10	10	2,901	135	-0,6682 to 2,621	0,7636
Crestal Mesial 2 mm vs. 5 mm Mesial 2 mm	1,096	-0,1400	1,236	10	10	3,671	135	-0,4091 to 2,880	0,3823
Crestal Mesial 1 mm vs. 2.5 mm Mesial 1 mm	1,736	0,3705	1,365	10	10	4,056	135	-0,2795 to 3,010	0,2252
Crestal Mesial 1 mm vs. 5 mm Mesial 1 mm	1,736	0,2830	1,453	10	10	4,316	135	-0,1920 to 3,097	0,1478
Crestal Central vs. 2.5 mm Central	2,183	0,9670	1,216	10	10	3,611	135	-0,4292 to 2,860	0,4106
Crestal Central vs. 5 mm Central	2,183	0,4140	1,769	10	10	5,254	135	0,1238 to 3,413	**0,0224**
Crestal Distal 1 mm vs. 2.5 mm Distal 1 mm	1,947	1,079	0,8685	10	10	2,580	135	-0,7763 to 2,513	0,8840
Crestal Distal 1 mm vs. 5 mm Distal 1 mm	1,947	0,5956	1,351	10	10	4,015	135	-0,2934 to 2,996	0,2396
Crestal Distal 2 mm vs. 2.5 mm Distal 2 mm	1,402	0,9295	0,4722	10	10	1,403	135	-1,173 to 2,117	0,9996
Crestal Distal 2 mm vs. 5 mm Distal 2 mm	1,402	0,5150	0,8866	10	10	2,634	135	-0,7581 to 2,531	0,8670
2.5 mm Mesial 2 mm vs. 5 mm Mesial 2 mm	0,1191	-0,1400	0,2591	10	10	0,7698	135	-1,386 to 1,904	>0,9999
2.5 mm Mesial 1 mm vs. 5 mm Mesial 1 mm	0,3705	0,2830	0,08751	10	10	0,2600	135	-1,557 to 1,732	>0,9999
2.5 mm Central vs. 5 mm Central	0,9670	0,4140	0,5530	10	10	1,643	135	-1,092 to 2,198	0,9978
2.5 mm Distal 1 mm vs. 5 mm Distal 1 mm	1,079	0,5956	0,4829	10	10	1,435	135	-1,162 to 2,128	0,9995
2.5 mm Distal 2 mm vs. 5 mm Distal 2 mm	0,9295	0,5150	0,4145	10	10	1,231	135	-1,230 to 2,059	>0,9999
